# A Case of Chronic Arthritis

**Published:** 1884-05

**Authors:** J. T. Kent

**Affiliations:** St. Louis


### CLINICAL BUREAU.

### A CASE OF CHRONIC ARTHRITIS.

PROF. J. T. KENT, A. M., M. D., ST. LOUIS.

Mrs. N., æt. about thirty-eight, has for about ten years been an invalid as a result of chronic arthritis of the left knee. When it was in the acute state she was treated by Dr. Hammer, a well-known St. Louis surgeon. It was cupped and blistered but the disease progressed. Continuously she was treated by the best allopathic surgeons and still it progressed. The last to have control of it was our lamented Dr. Hodgen, who placed it in a splint, saying that if anchylosis could not be accomplished it must come off. "A stiff leg or no leg," was his language. Two months in a splint failed to accomplish anchylosis.

July 16th, 1881, I was called to the case. The knee was painful and extremely sore to touch, enlarged to twice the size of the well one and very hard. The thigh was emaciated and the ankle and feet were œdematous. The limb was wrapped and she was in bed. She could sit up but the limb could not be moved much, it was so painful from motion. There was great burning in the soles and top of the head. Sulph.$^{55000}$ (Fincke) one dose dry. Sac. lac.

The husband came to me the next morning, saying that Mrs. N. was much worse. She had suffered greatly during the night and had pain all over the body. I visited her and urged her to bear her suffering, that it would pass off soon. She took Sac. lac. till August 20th, and Sulph.$^{81m}$ was given, one dose dry. Slight aggravation followed, but she said she could bear it, as the first medicine which aggravated had been followed by such relief. September 1st. The pain has all subsided and she is moving about the house on crutches. September 20th she sent for me. I found crape on the door, and learned that her husband had been sick a week and had died under allopathic treatment; that she had been up night and day attending him and was very nervous and the limb was much more painful. She took Ignatic for some days until the sad occasion had passed over a little, when I again paid my attention to the knee. October 8th she took Sulph.$^{81m}$ (Fincke), and she thought it gave her rest, but not much improvement in the knee. She continued Sac. lac. to November 12th. The joint has grown smaller, the foot is not so œdematous, no burning in the soles or top of head. Her appetite is good and she is gaining strength. In a general way she is much improved. Not seeing how matters could be improved by medicine, without better indications, I concluded to continue Sac. lac.

December 3d.—She complained of cold feet and that every change in the weather from warm to cold gave her pain in the knee and she had a *craving for eggs*. She had difficulty in keeping warm. Calc.$^{85m}$ (Fincke) and Sac. lac. for a month.

January 7th, 1882.—Feeling very comfortable; slept well most of her nights; feet warm, and there was not much pain in the knee; swelling in knee going down; she is about the house on crutches; the sensitiveness is gradually going out of the knee. Sac. lac.

During all this time there has been limited motion in the limb, but the slightest motion has always caused pain. But she has been able to swing it off the bed, holding the foot up to prevent flexion and then with her crutches she has been going about the house with comparative comfort.

February 3d.—Calc.$^{85m}$. Improving slowly.

March 25th.—There is some motion in the knee without much pain; the joint is slowly growing smaller; no swelling of the foot; she now wears a shoe that mates the right, the first time for ten years or more. Sac. lac.

April 4th.—No new symptoms; improving has ceased. Calc. c.$^{85m}$ and Sac. lac.

May 3d.—No change from last date; no new symptoms; eating well, sleeping well; countenance looks well. What shall I do? Prescribe for the knee? No, I wait. Sac. lac.

June 3d.—Sour eructations that seem to burn the pharynx but do not come up into the mouth; knee more painful; nights restless; must move about, which seems to relieve; drawing pain in the knee; gnawing pain in the stomach. "*A sour eructation, the taste of which does not remain in the mouth, but the acid gnaws in the stomach.*" *Lyc.* "*Incomplete burning eructations which only rise into the pharynx, where they cause a burning for several hours.*" (Allen.) Lyc.

Lycopodium having all the rest of the symptoms, it was given 71m, and Sac. lac. The knee became very painful and she was compelled to keep her bed for several days. Each day I visited her and she took Sac. lac.

July 2d.—She is walking with crutches and has very little pain in the knee; no pain in stomach or eructation. Improving.

August 3d.—Improving. Sac. lac.

September 2d.—Lycop.$^{71m}$ and Sac. lac.

September 6th.—Slight aggravation from the Lyc. Improving.

October 1st.—Improving. Sac. lac.

November 8th.—Improving. Sac. lac.

December 15th.—Lycopod.$^{71m}$ and Sac. lac.

January, 1883.—It is now eighteen months since taking this case. The patient is in good flesh, and the knee is the only thing that gives her trouble. There is still limited motion. The motion is not much painful except when forced flexion is attempted. She goes about the yard and out into the road. I furnished her a cane and advised laying aside one of the crutches. She has no fear of the knee being hit, which heretofore has been a great factor in the case.

May 1.—She walks with a crutch and cane. Limbs gaining motion continuously. No new symptoms. Knee nearly natural. She can bear some weight on the left foot. Lyc.$^{71m}$ dry and Sac. lac.

July 8th.—Rheumatic pains in both knees and such restlessness that she moves all night. Stiffness in joints, which passes off by motion; while in motion she feels better. Rhus tox.$^{1m}$ in water every three hours.

July 10th.—Improved. Restlessness all gone. Stiffness some better. Sac. lac.

August 5th.—Improving. Rhus tox.$^{32m}$ (Fincke), one dose, and Sac. lac.

September 1st.—I found her walking with one cane. She moved over the house to show me how well she could walk.

October 1st.—Improving. Rhus tox.$^{32m}$ one dose, and Sac. lac.

November 8th.—Rhus tox.$^{32m}$ one dose, and Sac. lac.

December 5th.—She walked with the aid of her cane two blocks to a street car, and came to my office from a far part of the city; she walked in my office without the aid of the cane.

January 7th, 1884.—Came to my office. She walks with a limp. Limited motion in the knee, but the soreness has gone. I asked her if she regretted going under constitutional treatment, to which she answered: "Ten thousand times, no!"

I have referred to two distinguished allopathic attendants, simply to show that the best surgical skill had been applied, and that the value of the purely homœopathic method may be the better appreciated. Ten years she grew worse, and in two and one-half years she was cured. If it be argued that she recovered without medicine, then the means that had been used were destroying her life.

